# The Respiratory Phenotype of Pompe Disease Mouse Models

**DOI:** 10.3390/ijms21062256

**Published:** 2020-03-24

**Authors:** Anna F. Fusco, Angela L. McCall, Justin S. Dhindsa, Lucy Zheng, Aidan Bailey, Amanda F. Kahn, Mai K. ElMallah

**Affiliations:** Department of Pediatrics, School of Medicine, Duke University, Durham, NC 27707, USA; anna.fusco@duke.edu (A.F.F.); angela.mccall440@duke.edu (A.L.M.); justin.dhindsa@duke.edu (J.S.D.); lucy.zheng1@duke.edu (L.Z.); aidan.bailey@duke.edu (A.B.); amanda.kahn@duke.edu (A.F.K.)

**Keywords:** pompe disease, breathing, respiratory

## Abstract

Pompe disease is a glycogen storage disease caused by a deficiency in acid α-glucosidase (GAA), a hydrolase necessary for the degradation of lysosomal glycogen. This deficiency in GAA results in muscle and neuronal glycogen accumulation, which causes respiratory insufficiency. Pompe disease mouse models provide a means of assessing respiratory pathology and are important for pre-clinical studies of novel therapies that aim to treat respiratory dysfunction and improve quality of life. This review aims to compile and summarize existing manuscripts that characterize the respiratory phenotype of Pompe mouse models. Manuscripts included in this review were selected utilizing specific search terms and exclusion criteria. Analysis of these findings demonstrate that Pompe disease mouse models have respiratory physiological defects as well as pathologies in the diaphragm, tongue, higher-order respiratory control centers, phrenic and hypoglossal motor nuclei, phrenic and hypoglossal nerves, neuromuscular junctions, and airway smooth muscle. Overall, the culmination of these pathologies contributes to severe respiratory dysfunction, underscoring the importance of characterizing the respiratory phenotype while developing effective therapies for patients.

## 1. Introduction

Pompe disease is a rare glycogen storage disease caused by an autosomal recessive mutation resulting in deficiency of acid α-glucosidase (GAA), the enzyme responsible for breaking down lysosomal glycogen [1,2]. As a result of this enzymatic deficiency, glycogen accumulates in the central nervous system and in cardiac, skeletal, and smooth muscle. Additionally, glycogen accumulation affects cellular processes, such as autophagy and metabolism [3,4,5]. Prior to the recent addition of Pompe disease to newborn screening, the incidence of Pompe disease was reported as 1:40,000 [6,7]. However, because newborn screening increased the accuracy of diagnosing Pompe disease, the reported frequency is now much higher, ranging from 1:27,800 to 1:8700 [8]. Disease severity, age at symptom onset, and disease progression vary widely depending on the mutation, level of residual functional enzyme present, and other modulating factors that have not been completely elucidated [9,10]. 

Patients are broadly characterized into two groups: infantile-onset Pompe disease (IPD) or late-onset Pompe disease (LOPD) [6,9,10]. Patients with IPD experience the most severe symptoms with onset in the first few months of life [6]. IPD patients have less than 1% of normal, functional GAA and do not survive beyond two years if untreated [4,9,11,12]. Cardiomegaly and respiratory insufficiency are often the first signs of IPD, which eventually progress to cardiorespiratory failure [3,13,14]. LOPD patients typically present in adolescence or adulthood, but cases can present as early as the first year of life. Patients with LOPD typically maintain 1%–20% functional GAA [9], however, GAA activity levels of 30% [15] to 40% [3,16] of normal have been reported in Pompe patients [3,16]. Glycogen accumulation in the respiratory system causes progressive respiratory insufficiency, forcing approximately 75% of children and 33% of adults with Pompe disease to rely on mechanical ventilation [17,18,19]. 

Enzyme replacement therapy (ERT) is the most common treatment for Pompe disease and involves the administration of recombinant human GAA (rhGAA). ERT improves survival by clearing glycogen in cardiac muscle and improving cardiac function. However, because ERT does not effectively clear glycogen in respiratory skeletal muscle, airway smooth muscle, and neural control centers, many patients on ERT still suffer from respiratory dysfunction and require ventilatory support [6,8,20,21,22,23,24,25]. Recent advances in gene therapy hold promise for treating Pompe disease, as gene therapy has the potential to ameliorate respiratory insufficiency [6,8,26,27,28,29,30]. However, before treating the respiratory pathology, thorough characterization of the respiratory phenotype in Pompe disease is necessary in order to develop precise, novel therapies.

Animal models have proven useful for studying the pathophysiology of Pompe disease. There are naturally occurring models of Pompe disease in certain breeds of cattle, dogs, quail, cats, and sheep [7,31,32,33,34,35,36,37]. Researchers have developed mice models of Pompe disease by disrupting exons 6 and 13. Mice models have greater phenotypic accuracy and are more convenient for laboratory research [7,38]. The model developed by Bijvoet et al., in which exon 13 is disrupted, has a near complete absence of GAA in all tissues and displays progressive accumulation of lysosomal glycogen in cardiomyocytes, hepatocytes, and skeletal muscle fibers [39]. However, when assessed up until 9 months of life, this model did not have any muscle weakness, despite its severe deficiency of GAA [38,39]. Similarly, the model developed by Raben et al., in which exon 6 is disrupted, has progressive accumulation of lysosomal glycogen in the muscles and motor neurons but does not display obvious muscle wasting and weakness until 8–9 months of age [40]. Several researchers have back-crossed this *Gaa^−/−^* to pure 129SVE [41,42,43,44,45,46,47,48]. The *Gaa^−/−^* mouse on the pure 129SVE background is thought to have less severe respiratory deficits as compared to the *Gaa^−/−^* mouse on the mixed B6/129 background [42,45,49]. In addition to these global knockout mice, mice with tissue-specific expression of human GAA (hGAA) were created to isolate the impact of GAA deficiency in specific tissues on respiration [49,50]. Since the development of these different mouse models, characterization of the respiratory phenotype of Pompe disease is more feasible. This review seeks to summarize the pathology and dysfunction observed in the respiratory system of *Gaa^−/−^* mouse models. An understanding of the impact of the respiratory system in Pompe disease is important for future therapeutic developments. 

## 2. Results

### 2.1. Literature Search

**Figure 1:**
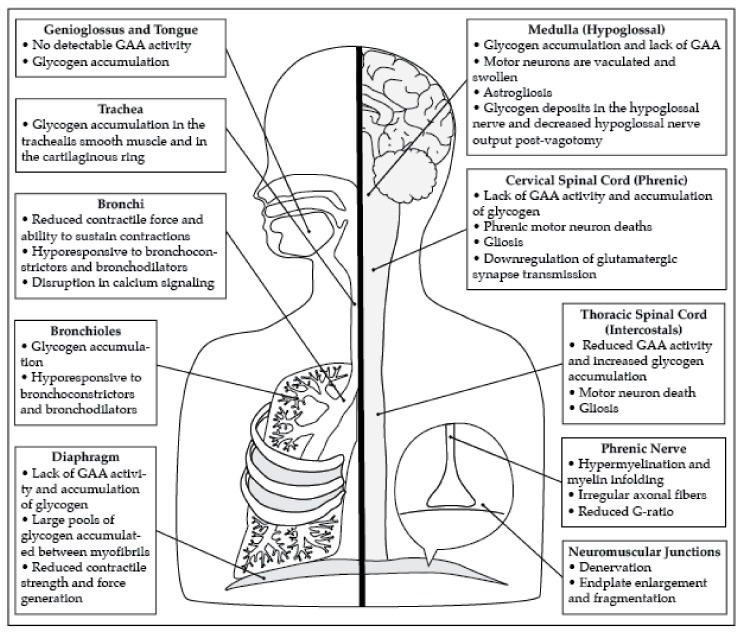
Pompe disease results in extensive pathology in both the muscular and neural components of the respiratory system. This figure summarizes the pathology present in the respiratory system that results in respiratory dysfunction.

The search terms identified 63 manuscripts. After using the exclusion criteria, a total of 27 manuscripts were included in this review. The manuscripts identified in this review describe pathology in the diaphragm, phrenic motor neurons, neuromuscular junctions, hypoglossal (XII) motor neurons, airway smooth muscle, and neural control centers (Figure 1). In addition, whole body plethysmography (WBP) in awake spontaneously breathing mice demonstrates that *Gaa^−/−^* mice have respiratory insufficiency. Neurophysiological recordings also demonstrate that *Gaa^−/−^* mice have blunted respiratory nerve output and abnormal respiratory rhythm. 

### 2.2. Diaphragm and Phrenic Motor Neuron Pathology

The diaphragm is the primary muscle of inspiration. Lysosomal glycogen accumulation in the diaphragm results in weakness and respiratory dysfunction. *Gaa^−/−^* mice on both the B6/129 and the pure 129SVE background lack GAA protein and GAA activity in the diaphragm [51,52,53,54,55]. As a result, the diaphragms of these *Gaa^−/−^* mice and the mice created by Bijvoet et al. have significant glycogen accumulation as demonstrated by positive periodic acid–Schiff (PAS+) staining and mass spectrometry [39,40,51,52,54,55,56,57,58,59,60]. Mass spectrometry provides evidence that *Gaa^−/−^* mice are born with glycogen accumulation in the diaphragm that progressively increases throughout life [56]. Accumulation of glycogen disrupts the structure of the skeletal muscle myofibrils that make up the diaphragm [39]. These structural abnormalities lead to a progressive decrease in the contractile strength of the diaphragm [45,49,61,62].

*Gaa^−/−^* mice have reduced GAA activity in motor neurons within the 3rd–5th segment of the cervical spinal cord where phrenic motor neurons are located [52,63,64]. Furthermore, PAS+ staining, a marker for glycogen accumulation, is present throughout the cervical spinal cord of *Gaa^−/−^* mice and is most evident in the ventral grey matter, where motor neurons, including phrenic motor neurons, are located [43,48,49,64]. In fact, PAS+ staining in the spinal cord reveals progressive accumulation of glycogen in the ventral horn of the spinal cord [65]. Phrenic motor neurons, identified by retrograde labeling with cholera toxin-B (CTb), exhibit significant pathology, such as vacuolization and swelling of somas [48,49]. In the cervical spinal cord, *Gaa^−/−^* mice have an overall reduction in motor neurons stained with choline acetyltransferase (ChAT) and, more specifically, a reduction of CTb-labeled phrenic motor neurons [49,52]. Particularly in late stages of the disease, *Gaa^−/−^* mice exhibit significant microgliosis and astrogliosis throughout the grey matter of the cervical spinal cord, as indicated by positive ionized calcium binding adaptor molecule 1 (IBA-1) and glial fibrillary acidic protein (GFAP), respectively [43,48,52]. The reduction of motor neurons and excessive glial activation is a hallmark of neurodegeneration and neuroinflammation [43,48]. 

Transcriptome analysis of the cervical spinal cord of *Gaa^−/−^* mice reveals significant alterations in mRNA expression in genes related to neurodegeneration, neuronal loss, neuroinflammation, signal transduction, synaptic plasticity, and cell metabolism [43]. *Gaa^−/−^* mice have increased mRNA expression associated with p53, apoptotic, and natural killer cell cytotoxicity pathways, suggesting neurodegeneration in the cervical spinal cord [43]. The mRNA expression changes are verified by positive terminal deoxynucleotidyl transferase dUTP nick and labeling (TUNEL) that shows DNA fragmentation induced by apoptosis and further suggests neurodegeneration [43]. Early in the disease progression, at around 6 weeks of age, there is no significant TUNEL staining in the ventral horn of the cervical spinal cord where the phrenic motor neurons are located [48]. By 6–8 months, when *Gaa^−/−^* mice begin to demonstrate respiratory dysfunction, there is significant TUNEL staining in the region of the phrenic motor nucleus. However, late in the disease progression, most *Gaa^−/−^* mice had positive TUNEL staining in cervical motor neurons in the region of the phrenic motor nucleus, however, one mouse had a reduction in motor neurons and only subtle TUNEL staining [48]. These results suggest that the presence of neurodegeneration changes throughout disease progression and that neurodegeneration in the phrenic motor nucleus is related to the decline in respiratory function. Changes in mRNA expression associated with signal transduction are also evident in the *Gaa^−/−^* mice cervical spinal cords. Pathways related to glutamatergic synaptic transmission are down-regulated. These pathways are essential for signaling in the respiratory synapses and could account for decreased respiratory nerve output in *Gaa^−/−^* mice [43].

Reduced phrenic nerve output could also be a result of pathology of phrenic motor neuron axons and their neuromuscular junctions within the diaphragm. The phrenic nerve of *Gaa^−/−^* mice have irregular fibers that are larger and have increased hypermyelination, myelin swelling, and myelin infoldings [46]. The phrenic nerves have a significantly lower G-ratio, or the ratio of the diameter of the axon to the thickness of the myelin [46]. *Gaa^−/−^* mice also have significant denervation of the diaphragm, as shown by the lack of overlap between presynaptic axon terminals and postsynaptic motor endplate terminals [46,64]. The diaphragm postsynaptic motor endplates of *Gaa^−/−^* mice are abnormally large as compared to wild type (WT) and have fragmented acetylcholine receptor clusters [46].

### 2.3. Pathology of the Tongue and Hypoglossal Motor Neurons

**Figure 2:**
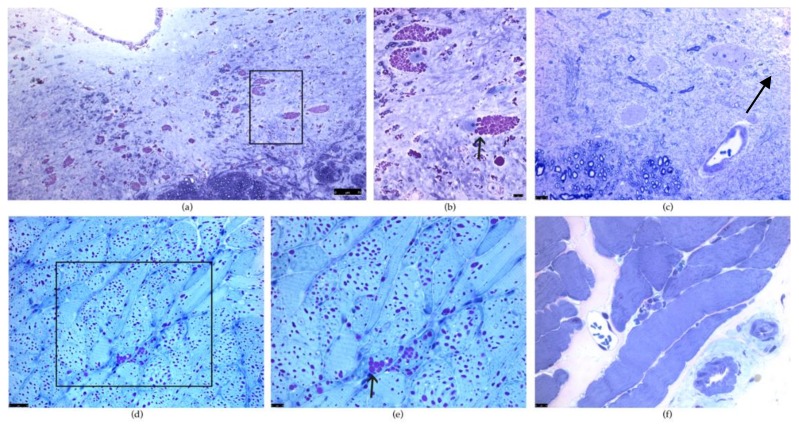
Periodic acid–Schiff (PAS) staining of the hypoglossal motor neurons and the tongue in *Gaa^−/−^* and wild type (WT) mice. (**a**,**b**): PAS staining is positive in the hypoglossal motor nucleus in *Gaa^−/−^* mice. (**b**) is a higher magnification of the boxed region (**a**) and illustrates PAS positive vacuoles indicative of glycogen-filled lysosomes in the hypoglossal motor neurons. Note the disruption in the architecture of the motor neuron resulting in displacement of the nuclei (arrow). (**c**) illustrates PAS staining in a WT mouse at the same magnification as (**b**). Note the lack of positive PAS staining in the XII motor neuron and the centrally placed nuclei (arrow). (**d**,**e**): PAS staining of a *Gaa^−/−^* tongue illustrates vacuoles filled with PAS positive glycogen (arrow). (**d**) is a higher magnification of the boxed region (**d**). (**f**): PAS staining of a WT tongue shows striated muscle with no evidence of PAS positive vacuoles. (**f**) is the same magnification as (**e**). Scale bars lower right in (**a**,**b**); lower left corner in (**c**,**d**,**e**,**f**). The scale bar denotes 7µm in (**a**), 10µm in (**b**,**c**,**e**,**f**), and 25µm in (**d**).

Maintaining a stable, open airway is important for proper respiratory function. The genioglossus muscle helps position the tongue during breathing and is important in maintaining upper airway patency during inspiration [66]. The hypoglossal nerves, which innervate the genioglossus, transmit an impulse to open the airway immediately before inspiration when the phrenic nerve innervates the diaphragm [66]. All *Gaa^−/−^* mice models have pathology in the tongue, particularly in the genioglossus muscle [39,42,47,51]. No detectable GAA activity is present in *Gaa^−/−^* mice tongues [47,51], which is accompanied by excessive glycogen buildup [39,42,47,51,64]. As in the phrenic motor neurons, the hypoglossal motor neurons have extensive pathology [42,44,66]. The hypoglossal motor neurons of *Gaa^−/−^* mice lack GAA protein [42,47] and have PAS+ staining [42,47,48,66]. Additionally, the hypoglossal motor neurons are also morphologically abnormal with swollen somas and glycogen-filled vacuoles [42,44,47] (Figure 2). PAS+ staining is also present in ependymal cells around the central canal as well as astrocytes in the hypoglossal motor pool [66]. Evidence of astrogliosis indicated by positive GFAP staining is also observed in the hypoglossal motor nucleus [64]. The pathology present in the tongue, genioglossus, and hypoglossal results in decreased airway patency and stability during inspiration. 

### 2.4. Pathology of Airway Smooth Muscle

Airway smooth muscle, such as tracheal and bronchial smooth muscle, plays a critical role in maintaining an open stable airway during inspiration and expiration. *Gaa^−/−^* mice have lysosomal glycogen accumulation in the tracheal and bronchial smooth muscle [39,67]. Excessive lysosomal glycogen in *Gaa^−/−^* tracheal and bronchial smooth muscle cells disrupts cellular architecture, causing nuclear displacement and severe cytoplasmic inclusion [67]. Following the administration of the bronchoconstrictive agent methacholine, *Gaa^−/−^* mice airway smooth muscle cells are hyporesponsive as evidenced by decreased overall respiratory resistance and central airway resistance [67]. The bronchial smooth muscle also has a reduced contractile force following exposure to methacholine, as measured by ex vivo bronchial ring isometric contraction [67]. The airway smooth muscle is also hyporesponsive to the bronchodilator albuterol [67]. Following the administration of albuterol to reverse the response to methacholine, the central airway resistance and bronchial force of the *Gaa^−/−^* mice did not decrease [67]. These results provide evidence that airway smooth muscle cells in *Gaa^−/−^* mice cannot properly contract or sustain contractions. This airway smooth muscle pathology is most likely a result of morphological pathology as well as calcium signaling disruption. Although intracellular calcium ([Ca^2+^]_i_) release from the bronchial smooth muscle to initiate a contraction is normal and the [Ca^2+^]_i_ reaches the same peak as wild type controls, the sustained [Ca^2+^]_i_ is significantly lower [67]. The dysfunction in the airway smooth muscle results in decreased airway patency and stability, which can exacerbate respiratory insufficiency. 

### 2.5. The Intercostal Muscles and Thoracic Motor Neuron Pathology

The intercostal muscles are a primary muscle group used during both inspiration and expiration. *Gaa^−/−^* mice experience pathology in the motor neurons that control the intercostal muscles. Lower GAA activity and PAS+ staining occur in the ventral grey matter of the 5th to 8th thoracic spinal segments, specifically within the region of the intercostal motor pool [47,48,63]. In 6 week old *Gaa^−/−^* mice, IBA-1 and GFAP staining already indicate evidence of gliosis and thus neuroinflammation. At 6 weeks, mice do not have positive TUNEL or cleaved caspase 3 (CC3) staining, which are both markers for apoptosis, indicating there is not yet neurodegeneration in the region of the intercostal motor pool [48]. However, at 10 months of age, *Gaa^−/−^* mice have a significant reduction in ChAT-positive motor neurons relative to WT in the region of intercostal motor pool and still have a significant increase in IBA-1 and GFAP positive glial cells with activated morphology [48,52]. Thus, it is possible neuroinflammation is prefacing neurodegeneration in the intercostal motor pools. Neurodegeneration and neuroinflammation in putative intercostal motor neurons could result in decreased signaling to the intercostal muscles and thus dysfunction during expiration and inspiration during respiratory challenge, similar to the respiratory insufficiency caused by Pompe disease.

### 2.6. Additional Neural Control Centers

Higher order neural control centers in the brain stem control respiratory motor output, rhythm generation, and respiratory reflexes. The nucleus of the solitary tract (NTS) is the primary integration site of afferent input regarding cardiorespiratory function. Information received by the NTS is sent to other respiratory control centers in the brainstem [66]. *Gaa^−/−^* mice have glycogen accumulation in the NTS [66]. In addition, positive CC3 and TUNEL staining is present in the NTS of *Gaa^−/−^* mice, indicating cells in the NTS are undergoing apoptosis. Positive GFAP staining also provides evidence of gliosis, which suggests neuroinflammation. The neurodegeneration and neuroinflammation in the NTS could result in dysfunction in the integration of afferent signaling [48] and impaired vagal reflexes observed in hypoglossal and phrenic nerve recordings of *Gaa^−/−^* mice [66]. Vagotomy normally causes a robust increase in phrenic and hypoglossal burst amplitude [66]. However, in *Gaa^−/−^* mice, the phrenic and hypoglossal burst amplitudes do not increase following vagotomy [66]. The dorsal vagal motor nucleus is negative for both CC3 and TUNEL staining and the motor neurons in the dorsal vagal motor nucleus do not have the characteristic enlarged somas and vacuolization seen in motor neurons in affected respiratory centers [44,48]. The lack of pathology in the dorsal vagal motor nucleus suggests that the lack of vagal reflexes is probably a result of compensation for NTS dysfunction in vagal intact mice [48,66].

The nucleus ambiguus of *Gaa^−/−^* mice, which houses the motor neurons controlling the pharyngeal and laryngeal muscles, contains PAS+ staining [66]. The motor neurons of the nucleus ambiguus of *Gaa^−/−^* mice have vesicle accumulation leading to enlarged somas compared to WT controls [44]. 

Finally, there are also abnormalities in other respiratory control centers in the brainstem, specifically in the pre-Botzinger complex and the noradrenergic neurons in the A1/C1 group of the ventral medulla [44]. Neurons in the pre-Botzinger complex, which are responsible for generating respiratory rhythms, experience vesicle accumulation in the somas of *Gaa^−/−^* but not WT mice [44]. Neurons in the A1/C1 group, which are speculated to modulate and activate breathing, especially during hypoxia, also have abnormal morphology and vesicle accumulation in *Gaa^−/−^* but not WT mice [44,68,69]. 

### 2.7. Respiratory Pathophysiology in the Gaa^−/−^ Mouse Model

Whole body plethysmography (WBP) and neurophysiological nerve recordings in Pompe mouse models reveal significant respiratory pathophysiology. WBP quantifies minute ventilation, lung volumes, and changes in flow in awake, spontaneously breathing mice [44,51,70]. WBP studies confirm that mouse models of Pompe disease have respiratory dysfunction [42,45,49,51,52,61,63,71,72]. Although results of WBP at baseline vary, most studies found that *Gaa^−/−^* mice have abnormal parameters of WBP. While breathing room air (FiO_2_ 0.21; N_2_ balance), these mice have greater expiratory time (Te) [45] and lower tidal volume (TV) [49,63], frequency (f) [45,49], tidal volume to inspiratory time ratio (TV/Ti) [49,61], minute ventilation (VE) [49], minute ventilation to expired CO_2_ ratio (VE/V_CO2_) [49,61], peak inspiratory flow (PIF) [49,61], and peak expiratory flow (PEF) [49,71]. Furthermore, during a hypercapnic challenge (FiCO_2_: 0.07; FiO_2_ 0.21; nitrogen balance), *Gaa^−/−^* mice on both the B6/129 and the 129SVE backgrounds have a reduced response to respiratory challenge [42,49,51,61,62,63,71,72]. For example, these mice have lower VE, TV, and PIF relative to WT during hypercapnic challenge, although these factors did increase relative to baseline [42,49,51,61,62,63,71]. The respiratory cycle in *Gaa^−/−^* mice also has higher variability than WT [66]. These abnormalities are predictive of pathology in inspiratory and expiratory muscles, upper and lower airway smooth muscles, and respiratory neural control centers. Lower VE and VE/V_CO2_ in *Gaa^−/−^* mice compared to WT mice suggest that the Pompe mice are hypoventilating [49]. In addition, *Gaa^−/−^* mice have decreased partial pressure of oxygen (PaO_2_), providing further evidence of hypoventilation under normoxia [49]. Interestingly, *Gaa^−/−^* mice also have higher hematocrit and hemoglobin levels, which may be an attempt to compensate for decreased oxygenation [49]. 

Neurophysiology is utilized to assess respiratory nerve output. The goal of neurophysiology is to record phrenic (diaphragm) and hypoglossal (tongue) motor output across a range of levels of respiratory drive [42,66,73]. Neurograms of hypoglossal and phrenic nerves show dysfunction in hypoglossal and phrenic efferent nerve output [49,66]. Hypoglossal neurograms reveal a double bursting pattern in the hypoglossal nerves of *Gaa^−/−^* mice [66]. Although there is no significant difference in frequency in *Gaa^−/−^* mice compared to WT, there is increased respiratory variability [66]. The amplitude of the hypoglossal nerve signal is similar between *Gaa^−/−^* mice and WT prior to vagotomy. However, following vagotomy, the *Gaa^−/−^* mice have significantly lower amplitudes [66]. Normally, after vagotomy, the hypoglossal and phrenic amplitudes significantly increase. Although the WT hypoglossal and phrenic burst amplitudes increase after vagotomy, the *Gaa^−/−^* hypoglossal and phrenic burst amplitudes do not significantly increase [66]. Furthermore, there is no pre-inspiratory hypoglossal activity in *Gaa^−/−^* mice. Whereas the hypoglossal nerve normally bursts before the phrenic to open and stabilize the airway before inspiration, *Gaa^−/−^* mice do not have that pre-inspiratory airway stabilization, which could result in problems stabilizing the upper airway, especially during increased respiratory drive [66]. Phrenic nerve efferent output is decreased in *Gaa^−/−^* mice shown by a reduced phrenic inspiratory burst amplitude, frequency, and slope of integrated inspiratory burst relative to WT mice [49,66].

To further confirm the importance of neuropathology in Pompe disease, measures of overall respiratory function were performed in *Gaa^−/−^* mice that have muscle-specific expression of hGAA, isolating the respiratory dysfunction resulting from neural pathology [49]. These mice have similar diaphragm contractile force as WT mice, confirming that the diaphragm muscle is functioning properly [49]. However, despite proper function of the diaphragm, *Gaa^−/−^* mice with muscle-specific correction of GAA expression still have respiratory dysfunction demonstrated by WBP. At baseline, the *Gaa^−/−^* mice and the *Gaa^−/−^* mice with GAA activity in the muscle have similar minute ventilations. However, during a hypercapnic challenge, the *Gaa^−/−^* mice with muscle-specific correction of GAA expression have a minute ventilation that is between the *Gaa^−/−^* mice and WT [49]. In addition, phrenic nerve recordings reveal that the *Gaa^−/−^* mice with GAA expression in the muscles have reduced phrenic inspiratory burst amplitude, frequency, and slope of integrated inspiratory burst relative to WT [49]. These results confirm that when the muscle is spared and produces GAA, respiratory deficits are still evident. This underscores the importance of the neural involvement in the respiratory dysfunction of Pompe disease mouse models. 

## 3. Discussion

This review describes the extensive respiratory pathology in the Pompe disease mouse model. The pathology in the *Gaa^−/−^* mouse model offers insight into the mechanisms of respiratory dysfunction in patients with Pompe disease. Respiratory dysfunction involves upper airway as well as respiratory muscle and motor neuron pathology. Similar to Pompe mice, patients with Pompe disease demonstrate respiratory pathophysiology and insufficiency, as well as upper airway pathology [74,75,76,77,78,79]. Furthermore, IPD and LOPD patients have reduced vital capacity [76,80,81], which is reminiscent of the reduced tidal volume during the respiratory challenge demonstrated by WBP in mice [51,62,63,71]. 

Although respiratory dysfunction was traditionally attributed to muscle pathology and weakness, recent evidence in mice and humans indicate pathology in both respiratory muscles and the neurons that control those muscles [44,49,82]. Accumulation of glycogen is seen throughout respiratory muscles and control neurons. In addition, mouse models that have muscle-specific expression of hGAA still have respiratory dysfunction [49], underscoring the importance of neuronal involvement in Pompe disease and demonstrating that neuronal pathology and dysfunction is not solely a result of muscle pathology. Therefore, the currently available ERT treatment with rhGAA may not effectively target neural tissue [83] because rhGAA cannot cross the blood–brain barrier. Furthermore, although ERT has significantly improved survival and quality of life of patients with Pompe disease, some patients on ERT still have mild to moderate accumulation of glycogen in the diaphragm [84], intercostal muscles [84], and airway smooth muscle [25,85] and pathology in respiratory control neurons [85]. Therefore, novel therapies need to target both muscle and neuronal pathology in order to treat the respiratory dysfunction. 

During inspiration, the primary muscle in use is the diaphragm, which is controlled by phrenic motor neurons. The diaphragm of Pompe mice lack GAA activity and have accumulation of glycogen that disrupts the structure of the myofibrils and results in muscle weakness [39,40,45,51,52,56,57,86,87]. Similarly, post-mortem studies of patients with both IPD and LOPD reveal glycogen accumulation in the diaphragm [84,88,89,90]. In some patients, there is severe disruption of the diaphragm myofibrils with degeneration and large vacuoles of glycogen evident on post-mortem studies [89,90,91]. Further evidence for diaphragm weakness is seen in LOPD patients who have decreased trans-diaphragmatic twitch pressure after magnetic phrenic nerve stimulation [92]. Diaphragm pacing improves ventilation in patients with LOPD and may also provide some targeted rehabilitation to the phrenic motor neurons [92]. 

Glycogen accumulation in the phrenic motor neurons results in neurodegeneration and denervation of the diaphragm [43,46,48,49,52,63,64,66]. In addition, the phrenic nerve of Pompe mice has irregular axon fibers, abnormal myelination, and a lower G-ratio [46]. Furthermore, the neuromuscular junctions of Pompe mouse models have irregular distribution of acetylcholine receptor clusters as well as limited colocalization of presynaptic and post synaptic labels [46,64]. Similarly, post-mortem studies of LOPD and IPD patients reveal glycogen accumulation and swelling in neurons in the anterior horns of the spinal cord [49,84,86,93,94,95]. In addition, post-mortem phrenic nerve analysis in an LOPD patient case revealed abnormal glycogen accumulation in Schwann cells [88]. 

Neural control of upper airway muscles is also important for breathing. The hypoglossal motor neurons help maintain a stable, open airway during breathing by controlling the genioglossus muscle [66]. Pompe mouse models display pathology in hypoglossal motor neurons and the hypoglossal nerve, as indicated by positive PAS staining, hypoglossal neurograms, and nerve burst amplitude [66] [42,44,47,48,66]. In addition, the tongue and specifically the genioglossus muscle in Pompe mice have glycogen accumulation [39,42,51]. Pompe patients have similar pathology in the genioglossus muscle and hypoglossal motor neurons as Pompe mice. The tongues of Pompe patients have glycogen accumulation, vacuolar myopathy, and atrophy [79,87,96]. Macroglossia, or enlargement of the tongue, is very common in patients with Pompe disease and can result in obstruction of the airway, especially during sleep [80,81,96]. Neurons in the hypoglossal motor nucleus of Pompe patients also have severe glycogen accumulation that exacerbates upper airway obstruction and hypoventilation [86,95]. 

The tracheal and bronchial airway smooth muscle are also significantly impacted in Pompe disease. As summarized above, there is significant glycogen accumulation in the bronchial and tracheal smooth muscle tissue of Pompe mice, resulting in reduced airway patency [67]. Similarly, studies of IPD and LOPD patients show glycogen accumulation in the trachea, bronchi, and bronchioles, which leads to trachea-bronchomalacia and an inability to maintain an open airway [25,84,85]. This malacia exacerbates respiratory insufficiency since the smaller airway radius results in increased resistance to airflow. Finally, there is a lack of research studying pathology of the larynx, pharynx, amd nucleus ambiguus in Pompe patients, all of which are important for upper airway patency.

Higher order neural control centers coordinate breathing, generate the rhythm of breathing, and produce respiratory reflexes [44]. The pre-Botzinger complex is responsible for generating the rhythm of breathing. Pompe mice have abnormal vesicle accumulation in the pre-Botzinger complex. Although, the pre-Botzinger complex has not been studied in patients with Pompe disease, sleep studies reveal central sleep apneas that are suggestive of pathology in the respiratory rhythm generators [97]. 

Thus, respiratory motor pathology results from a spectrum of pathology of the motor units: the muscle, the neuromuscular junction, the nerves, and the motor neurons. It is difficult to discern whether the muscle phenotype results in a weak neuromuscular junction resulting in neuronal weakness or whether the motor neuron and nerve pathology contributes to this more. Based on the above mouse models, we know that at as early as 6 weeks of age there is significant neuronal pathology [43,48,98], and muscular pathology is seen as early as at 3 weeks of age [39,99]. Additionally, mice with muscle specific expression of hGAA do not have diaphragm pathology but do have neuronal pathology and respiratory insufficiency in between that of *Gaa^−/−^* mice and WT mice [49]. As a result, it is most likely that both contribute to the respiratory neuromuscular weakness and respiratory insufficiency. However, in humans, there appears to be more variability in the clinical course and the neurological respiratory motor neuron pathology is difficult to systematically examine. We are aware that some patients have a more rapid respiratory decline and some respond better to ERT [75,76], whereas others have significant pathology post-mortem despite ERT. It is unclear if these clinical differences are a result of the variable GAA genetic mutations that these patients carry. Future studies will need to evaluate the correlation between genetic mutations and respiratory muscle and neuronal pathology. However, overall, post-mortem studies show significant muscle and neuronal pathology and glycogen accumulation in both LOPD and IPD patients that died from respiratory distress. Therefore, we believe that both the muscle and neuronal pathology needs to be considered in the design of future therapies. 

In conclusion, Pompe disease causes pathology throughout the respiratory system, which without treatment can result in devastating respiratory dysfunction and eventual respiratory failure. The Pompe disease mouse model provides the opportunity to study the impact of novel therapies on respiratory function. Thus, a thorough understanding of the respiratory phenotype of Pompe disease will inform research into novel treatments so that they can completely address all aspects of the respiratory phenotype and improve quality and longevity of life. 

## 4. Materials and Methods 

**Figure 3:**
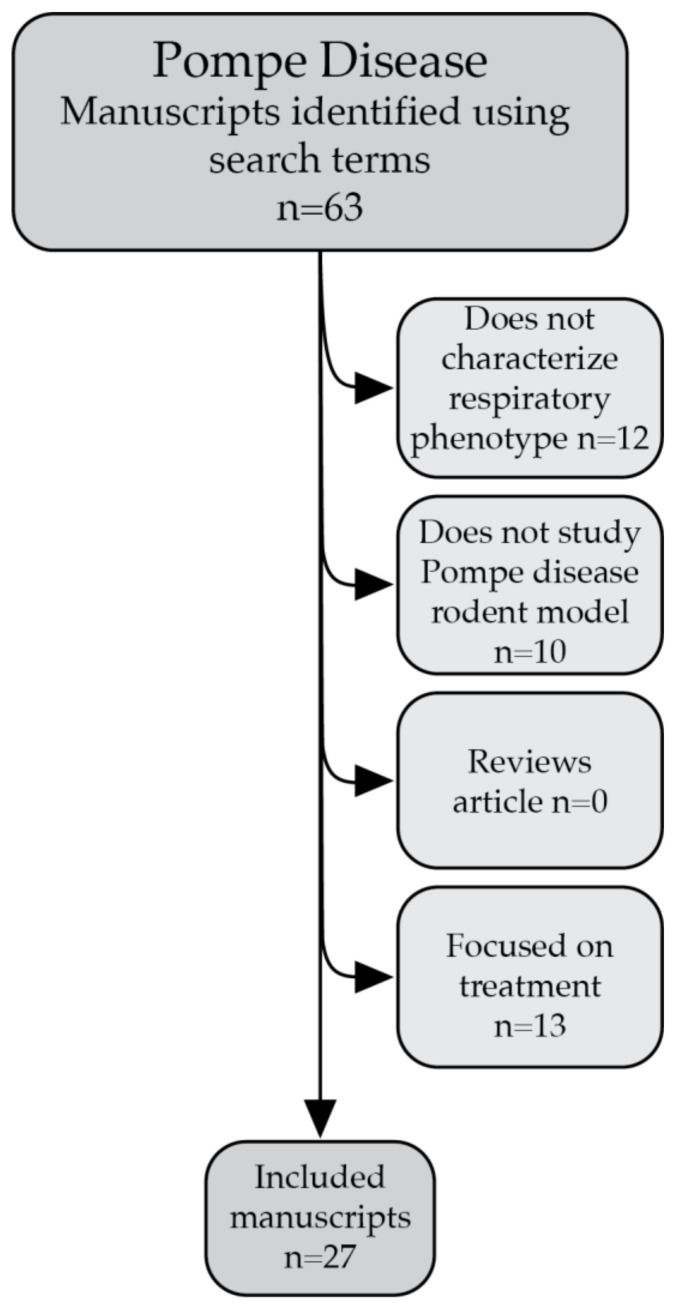
This figure shows the specific exclusion criteria used to find the final set of included manuscripts. Manuscripts were excluded if they did not characterize the respiratory phenotype, did not use rodent models of Pompe disease, were review articles, or focused on assessing the efficacy of treatments.

Manuscripts for this review were identified in PubMed using the search term (((((“Glycogen Storage Disease Type II” (MeSH)) OR (Pompe Disease(Title/Abstract) OR Type 2 Glycogen Storage Disease(Title/Abstract) OR Glycogen Storage Disease Type 2(Title/Abstract) OR Glycogen Storage Disease Type II(Title/Abstract) OR Acid Alpha Glucosidase Deficiency(Title/Abstract) OR Acid Maltase Deficiency(Title/Abstract)))) AND ((“Respiration” (MeSH)) OR (Breath*(Title/Abstract) OR Plethysmography(Title/Abstract) OR Diaphragm(Title/Abstract) OR Phrenic(Title/Abstract) OR Respiratory(Title/Abstract) OR PreBötzinger Complex(Title/Abstract) OR lung(Title/Abstract) OR trachea(Title/Abstract) OR Pharynx(Title/Abstract) OR Larynx(Title/Abstract) OR Apnea(Title/Abstract) OR Nucleus Ambiguus(Title/Abstract) OR Hypoglossal(Title/Abstract)))) AND ((“Mice” (MeSH)) OR (Mice[Title/Abstract] OR Mouse(Title/Abstract) OR Rat(Title/abstract) OR rodent(Title/Abstract) OR Murine(Title/Abstract)))) AND English(Language). Exclusion criteria were designed prior to reviewing the manuscripts. Manuscripts were excluded if they did not focus on characterizing the respiratory dysfunction and pathology in components of the respiratory system in Pompe rodent models. Review articles were also excluded. Articles focusing on the efficacy of a treatment were only included if, while doing so, dysfunction or pathology in the respiratory system were evaluated in untreated Pompe rodents and compared to an untreated WT control. Figure 3 shows how manuscripts were excluded and how many manuscripts were excluded using each exclusion criteria, leading to the final set of included manuscripts. Additional manuscripts were used to better explain techniques such as neurophysiology or whole body plethysmography and to explain the importance of different components of the respiratory system.

## 5. Conclusions

In conclusion, the respiratory pathology noted in the Pompe disease mouse models involves dysfunction of the diaphragm, neuromuscular junctions, phrenic and hypoglossal nerves and motor neurons, airway smooth muscle, accessory respiratory muscles, and various neurological control centers. Current literature describing pathology in Pompe disease models utilize *Gaa^−/−^* mice with mice of varying backgrounds. Characterization of the respiratory phenotype of Pompe disease mouse models is necessary to improve understanding of disease respiratory pathology, providing essential information for the development of novel therapies that address the shortfalls of current treatments and contribute to improved quality of life.

## Abbreviations

GAA	acid α-glucosidase
IPD	Infantile Onset Pompe Disease
LOPD	Late Onset Pompe Disease
ERT	Enzyme Replacement Therapy
rhGAA	recombinant human GAA
hGAA	human GAA
XII	Hypoglossal
WBPPAS+	Whole Body PlethysmographyPositive Periodic Acid-Schiff
CtB	Cholera Toxin B
ChAT	Choline Acetyltransferase
IBA-1	Ionized Calcium Binding Adaptor Molecule 1
GFAP	Glial Fibrillary Acidic Protein
TUNEL	Terminal Deoxynucleotidyl Transferase dUTP Nick and Labeling
WT	Wild Type
CC3	Cleaved Caspase 3
NTS	Nucleus of the Solitary Tract
Te	Expiratory Time
TV	Tidal Volume
Te	Expiratory Time
f	Frequency
TV/Ti	Ratio of Tidal Volume to Inspiratory Time
VE	Minute Ventilation
VE/VCO_2_	Ratio of Minute Ventilation to Expired CO_2_
PIF	Peak Inspiratory Flow
PEF	Peak Expiratory Flow
PaO_2_	Partial Pressure of Oxygen
